# Development and Validation of an 8-Gene Signature to Improve Survival Prediction of Colorectal Cancer

**DOI:** 10.3389/fonc.2022.863094

**Published:** 2022-05-10

**Authors:** Leqi Zhou, Yue Yu, Rongbo Wen, Kuo Zheng, Siyuan Jiang, Xiaoming Zhu, Jinke Sui, Haifeng Gong, Zheng Lou, Liqiang Hao, Guanyu Yu, Wei Zhang

**Affiliations:** Department of Colorectal Surgery, Changhai Hospital, Shanghai, China

**Keywords:** colorectal cancer, risk score, overall survival, recurrence-free survival, prognostic signature

## Abstract

**Background:**

Most prognostic signatures for colorectal cancer (CRC) are developed to predict overall survival (OS). Gene signatures predicting recurrence-free survival (RFS) are rarely reported, and postoperative recurrence results in a poor outcome. Thus, we aim to construct a robust, individualized gene signature that can predict both OS and RFS of CRC patients.

**Methods:**

Prognostic genes that were significantly associated with both OS and RFS in GSE39582 and TCGA cohorts were screened *via* univariate Cox regression analysis and Venn diagram. These genes were then submitted to least absolute shrinkage and selection operator (LASSO) regression analysis and followed by multivariate Cox regression analysis to obtain an optimal gene signature. Kaplan–Meier (K–M), calibration curves and receiver operating characteristic (ROC) curves were used to evaluate the predictive performance of this signature. A nomogram integrating prognostic factors was constructed to predict 1-, 3-, and 5-year survival probabilities. Function annotation and pathway enrichment analyses were used to elucidate the biological implications of this model.

**Results:**

A total of 186 genes significantly associated with both OS and RFS were identified. Based on these genes, LASSO and multivariate Cox regression analyses determined an 8-gene signature that contained ATOH1, CACNB1, CEBPA, EPPHB2, HIST1H2BJ, INHBB, LYPD6, and ZBED3. Signature high-risk cases had worse OS in the GSE39582 training cohort (hazard ratio [HR] = 1.54, 95% confidence interval [CI] = 1.42 to 1.67) and the TCGA validation cohort (HR = 1.39, 95% CI = 1.24 to 1.56) and worse RFS in both cohorts (GSE39582: HR = 1.49, 95% CI = 1.35 to 1.64; TCGA: HR = 1.39, 95% CI = 1.25 to 1.56). The area under the curves (AUCs) of this model in the training and validation cohorts were all around 0.7, which were higher or no less than several previous models, suggesting that this signature could improve OS and RFS prediction of CRC patients. The risk score was related to multiple oncological pathways. CACNB1, HIST1H2BJ, and INHBB were significantly upregulated in CRC tissues.

**Conclusion:**

A credible OS and RFS prediction signature with multi-cohort and cross-platform compatibility was constructed in CRC. This signature might facilitate personalized treatment and improve the survival of CRC patients.

## Introduction

Colorectal cancer (CRC) is the third most common cancer in the world and the second leading cause of cancer-related death (1). In the last few decades, a decreased incidence and improved prognosis have been achieved in CRC through accurate screening and comprehensive management, namely, surgical resection, chemoradiotherapy, and immunotherapy (2, 3). However, the survival of advanced CRC patients is still grim, especially for the 20–25% of patients with distant metastases at the diagnostic stage (4–6). For patients with surgical indications, early postoperative recurrence is pretty difficult to prevent, which is largely responsible for the poor prognosis (7). Therefore, there is a significant need to identify novel, reliable biomarkers for survival assessment and recurrence prediction in CRC management.

As CRC treatment has entered the era of precision medicine, many studies have endeavored to accurately evaluate patient survival in various ways. Traditional clinicopathological features, such as C-reactive protein (8), tumor size (9), and lymph node metastasis (10), have been proven to be independent prognostic factors in CRC. Nevertheless, due to the remarkably high genetic and genomic heterogeneity in CRC patients, these factors are not effective enough in terms of survival prediction (11). Recent studies suggest that the establishment of gene signatures based on large-scale gene expression datasets is a promising tool for survival assessment in various cancers (12, 13). As previously reviewed (14), multiple prognostic gene models with enormous clinical value have been established in the context of CRC. Intriguingly, these models are developed primarily to predict overall survival (OS), and few of them predict recurrence-free survival (RFS). Considering recurrence after surgery is a feature of CRC and it hinders long-term patient survival, RFS prediction is of considerable significance. Thus, it is essential to identify a reliable gene signature for both OS and RFS prediction.

As far as we know, only three gene signatures have previously predicted both OS and RFS, and the accuracy remains to be improved (15–17). In this study, we systematically analyzed the correlation between gene expression and OS or RFS of patients and established an 8-gene signature with enhanced performance for both OS and RFS prediction. The proposed model was superior to several previously reported models for predicting survival. Moreover, this signature was closely associated with DNA replication, cell division, and cell adhesion. These findings might provide valuable guidance for personalized treatment and optimal management of CRC patients.

## Methods

### Data Collection

Two public CRC cohorts with clinical and gene expression data were used for survival analyses in this study. Among them, the GSE39582 cohort (N = 536) was retrieved from the Gene Expression Omnibus (GEO, https://www.ncbi.nlm.nih.gov/geo/) database and used as the training set. The TCGA cohort (N = 368) was downloaded from the TCGA hub at UCSC Xena (https://tcga.xenahubs.net) and used for external validation.

In each cohort, patients with incomplete clinical information, OS time <1 month or RFS time <1 month were strictly excluded. Additionally, 72 formalin-fixed and paraffin-embedded CRC tissues and matched adjacent non-tumor tissues were collected at the Department of Colorectal Surgery at Shanghai Changhai Hospital. None of the patients received preoperative chemotherapy or radiotherapy. Written informed consent was obtained from all patients. This study was conducted and approved in accordance with the Declaration of Helsinki and the Ethics Committee of Shanghai Changhai Hospital.

### Construction of the 8-Gene Signature

To screen candidate genes for signature construction, univariate Cox regression analysis was first conducted to identify genes significantly associated with OS or RFS (p <0.05) in the GSE39582 and TCGA cohorts. Then, a Venn diagram (18) was employed to select common survival-related genes in these two cohorts. Credible prognostic genes were submitted to the Metascape database (19) for function annotation and pathway enrichment. Subsequently, they were submitted to the least absolute shrinkage and selection operator (LASSO) regression analysis and the following multivariate Cox regression analysis using OS events and time to generate an optimal risk signature with the minimum Akaike Information Criterion (AIC) value. Based on the expression level and the corresponding coefficient of each prognostic gene generated from the multivariate Cox regression analysis, the risk score of each sample was computed as follows: Risk score = (coefficient 1 ∗ expression value of gene 1) + (coefficient 2 ∗ expression value of gene 2) +… + (coefficient n ∗ expression value of gene n).

### Prognostic Performance of the 8-Gene Signature

Patients in each cohort were then assigned matched risk scores and they were divided into low- and high-risk groups based on the medium value of these risk scores. Kaplan–Meier (K–M) survival curves, univariate Cox analyses, and calibration curves were adopted to evaluate the prognostic performance of this signature. Time-dependent area under the curve (AUC) values were employed to compare the predictive accuracy of clinical predictors and the risk signature. Moreover, receiver operating characteristic (ROC) curves were used to compare the predictive ability of our signature with nine recently published prognostic signatures for CRC patients (20–28).

### Nomogram Construction in GSE39582 Training Cohort

The nomogram is a widely used method to quantitatively predict patient survival. To facilitate the clinical application of this signature, we established the nomogram based on prognostic variables derived from univariate Cox regression analysis in the GSE39582 training cohort to predict 1-, 3-, and 5-year survival probabilities. The predictive performance of the Nomogram was validated in the TCGA cohort through ROC curves.

### Functional Annotation and Pathway Enrichment of the 8-Gene Signature

To preliminarily clarify the underlying mechanism of the high risk score-resulted unfavorable prognosis, genes significantly correlated with risk scores (p <0.05) were identified by the Pearson correlation analysis in the GSE39582 and TCGA cohorts, respectively. A Venn diagram was applied to determine common correlated genes. These genes were then submitted to Gene Ontology-Biological Process (GO-BP) analysis and The Kyoto Encyclopedia of Genes and Genomes (KEGG) pathway enrichment analysis on the DAVID website (29, 30), respectively.

### Immunohistochemical (IHC) Staining

IHC assays were performed as previously reported (31). To quantify the expression of these molecules, IHC scores were determined by two independent observers using the index of H-Score. H-SCORE = ∑(PI × I) = (percentage of cells of weak intensity × 1) + (percentage of cells of moderate intensity × 2) + percentage of cells of strong intensity × 3), where PI indicates the proportion of positive signal pixel area; and I represents the coloring intensity. The final staining scores from two observers were averaged and rounded to the nearest whole number.

### Statistical Analysis

The statistical analyses and graphic study were conducted using R software (version 3.5.2). K–M survival curves with log-rank tests were executed by the ‘survival’ package. ROC analyses were plotted by the ‘survivalROC’ package. Time-dependent AUC values were generated using the ‘timeROC’ package. In Cox regression analyses, we estimated the hazard ratios (HRs) of CRC subgroups with standard clinicopathological variables: age at diagnosis (≥65 vs <65), gender, and tumor size (≥T2 vs <T2), lymph node invasion (≥N1 vs <N1), metastatic spread (M1 vs M0), disease stage (≥II vs <II), chemotherapy and resection margin (>R0 vs R0). Continuous risk scores were classified into low- and high-risk groups according to the medium value of their risk scores. Parameters in univariate and multivariate Cox analyses were generated from the ‘survival’ package and were visualized using the ‘forestplot’ package. LASSO regression analysis was conducted by the ‘glmnet’ package. The nomogram and calibration curves were produced by the ‘rms’ R package. Boxplots depicting the distribution of gene expression and risk scores were derived from the ‘ggpubr’ package. P <0.05 was considered significant.

## Results

### Construction of the 8-Gene Signature in the GSE39582 Training Cohort

**Figure 1:**
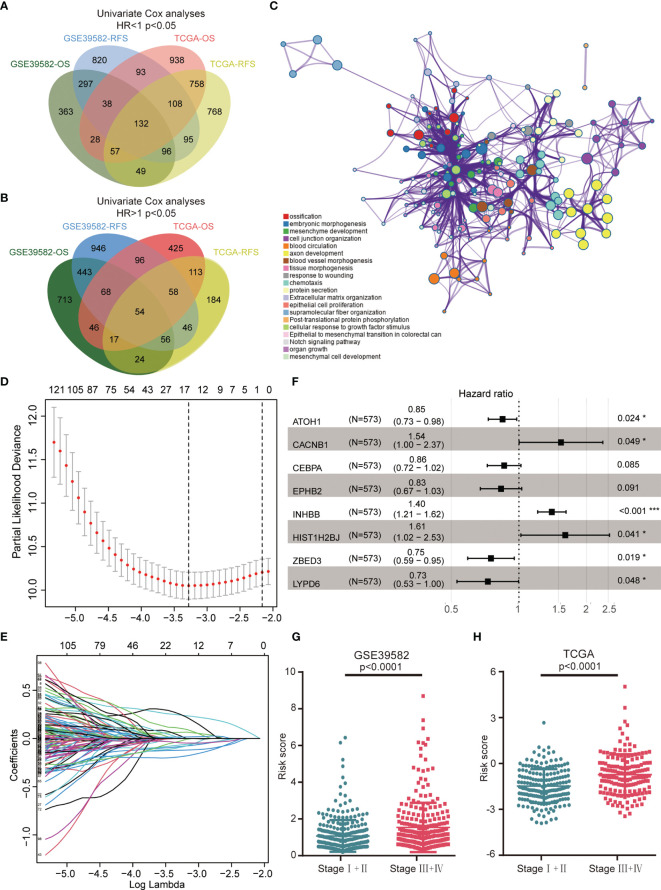
Construction of the 8-gene signature in the GSE39582 training cohort. **(A)** A Venn diagram screened 132 protective genes that were significantly associated with both OS and RFS in two CRC cohorts. **(B)** A Venn diagram identified 54 risky genes that were significantly related to both OS and RFS. **(C)** Enriched pathways of abovementioned 186 credible prognostic genes. **(D)** Cross-validation for tuning parameter (lambda) screening in the LASSO regression model. **(E)** LASSO coefficient profiles of 15 prognostic genes. **(F)** Forest plot of the eight genes. **(G)** Distribution of risk scores in different TNM stage of GSE39582 samples. **(H)** Distribution of risk scores in different TNM stage of TCGA samples.

**Figures 1A, B** illustrated the workflow of credible prognostic gene identification. A total of 132 protective genes (Hazard ratio <1, **Figure 1A**) and 54 risky genes (Hazard ratio >1, **Figure 1B**) were screened by univariate Cox regression analysis and a Venn diagram. Function annotation and pathway enrichment analyses jointly showed that these genes were primarily associated with epithelial–mesenchymal transition in colorectal cancer, the Notch signaling pathway, and multiple cancer-related pathways (**Figure 1C**). These 186 genes were subsequently subjected to LASSO regression analysis and 15 candidate genes with the most powerful predictive features were identified (**Figures 1D, E**). Following multivariate Cox regression analysis, the optimal 8-gene signature was finally selected in the avoidance of overfitting (**Figure 1F**). Based on the expression and matched coefficients of these eight genes, an individual risk score was calculated as follows: Risk score = −0.16788 ∗ expression value of ATOH1 + 0.431768 ∗ expression value of CACNB1 − 0.15152 ∗ expression value of CEBPA − 0.18303 ∗ expression value of EPPHB2 + 0.475006 ∗ expression value of HIST1H2BJ + 0.338153 ∗ expression value of INHBB − 0.31889 ∗ expression value of LYPD6 − 0.28645 ∗ expression value of ZBED3. Additionally, the risk scores were significantly higher in patients with a high TNM (Tumor, Node, Metastasis) stage in the GSE39582 and TCGA cohorts, suggesting that the 8-gene signature was associated with tumor progression (**Figures 1G, H**).

### Prognostic Performance of the 8-Gene Signature in the GSE39582 Training Cohort

**Figure 2:**
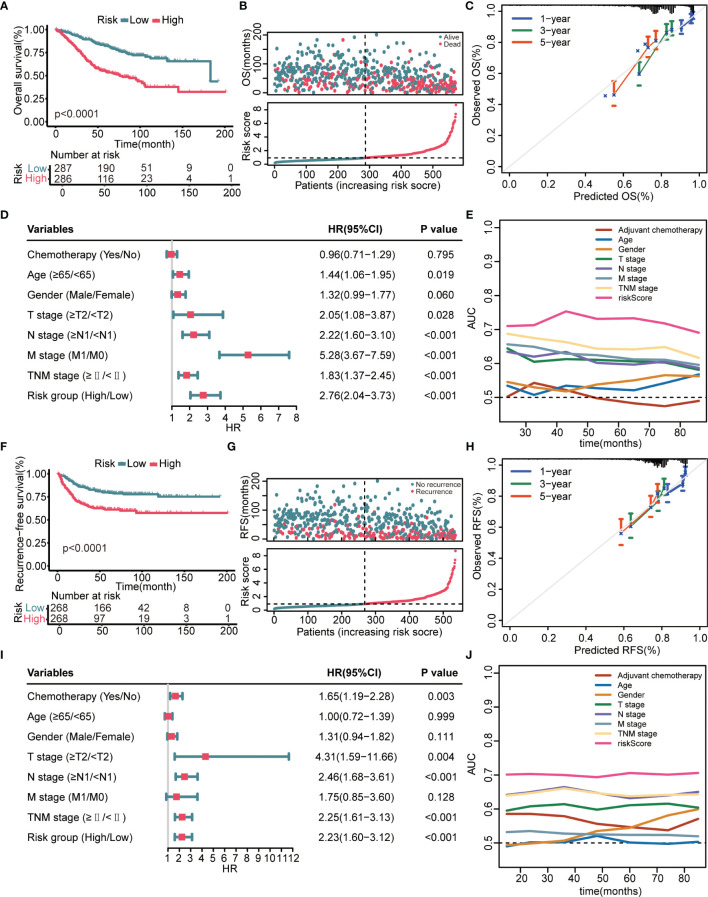
Prognostic performance of the 8-gene signature in the GSE39582 training cohort**. (A)** K–M curves evaluate the OS difference between low- and high-risk groups. **(B)** From top to bottom are the risk score distribution and overall survival status distribution. **(C)** Calibration curves reflect the accordance between observed OS and predicted OS. **(D)** Univariate Cox regression analysis examines prognostic roles of risk score and clinical features for OS. **(E)** Time-dependent AUC values illustrate the OS predictive accuracy of gene signature and clinical predictors over time. **(F)** K–M curves evaluate the RFS difference between low- and high-risk groups. **(G)** From top to bottom are the risk score distribution and recurrence-free survival status distribution. **(H)** Calibration curves represent the agreement between observed RFS and predicted RFS. **(I)** Univariate Cox regression analysis examines prognostic roles of risk score and clinical features for RFS. **(J)** Time-dependent AUC values show the RFS predictive accuracy of gene signature and clinical predictors over time.

K–M survival analysis demonstrated that patients in the high-risk group had a significantly decreased OS (**Figure 2A**). The distribution of the risk scores and overall survival status is illustrated in **Figure 2B**. The results demonstrated that patients with a low-risk score had a markedly decreased mortality rate compared with patients with a high-risk score. The calibration curves showed that the predicted OS by this signature was in good accordance with the observed OS (**Figure 2C**). The results of univariate Cox regression analysis suggested that this signature was an independent risk factor for OS (**Figure 2D**). Apart from the 8-gene signature, several clinical features, namely, age, T stage, N stage, and M stage, could also indicate unfavorable outcomes (**Figure 2E**). However, as shown in **Figure 2E**, the AUC values of the risk signature for OS prediction were higher than those of clinical features over time, indicating that this signature outperformed clinical predictors in prognosis assessment. In addition to OS, this model could also effectively stratify patients with different RFS. **Figures 2F, G** showed that patients in the high-risk group had remarkably decreased RFS time and elevated recurrence rate compared with patients in the low-risk group. Calibration curves showed that the predicted RFS by this signature agreed well with the observed RFS (**Figure 2H**). **Figures 2I, J** jointly proved that the 8 gene signature was a more effective RFS predictor.

### Prognostic Performance of the 8-Gene Signature in the TCGA Validation Cohort

**Figure 3:**
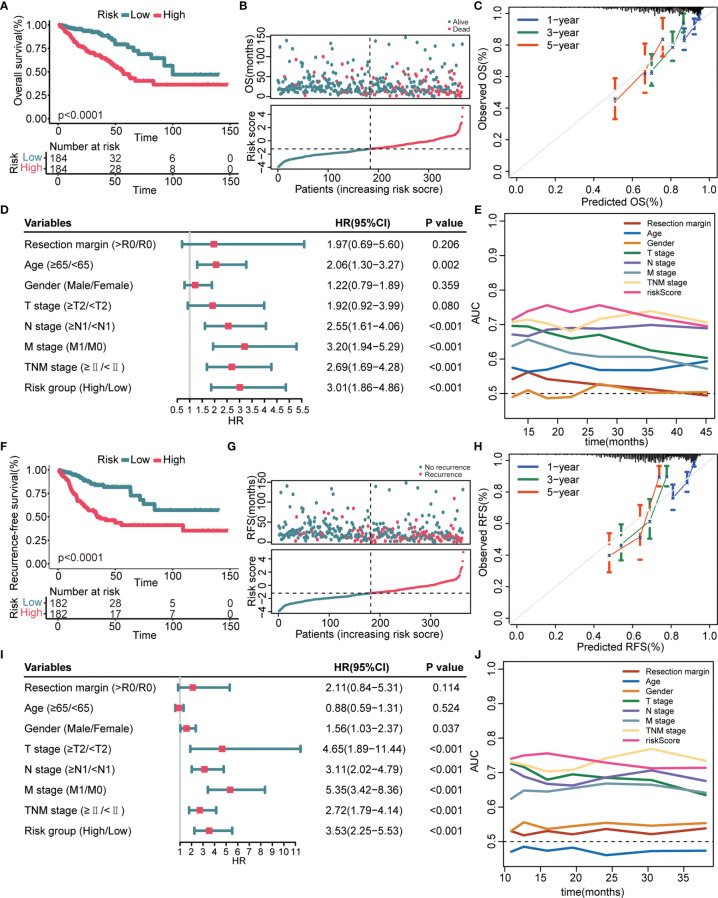
Prognostic performance of the 8-gene signature in the TCGA validation cohort**. (A, F)** K–M survival curves of OS **(A)** and RFS **(F)**, respectively. **(B, G)** From top to bottom are the risk score distribution and OS status distribution **(B)** or RFS status distribution **(G)**. **(C, H)** Calibration curves of OS **(C)** and RFS **(H)**, respectively. **(D, I)** Univariate Cox regression analysis identifies independent risk factors of OS **(D)** and RFS **(I)**, respectively. **(E, J)** Time-dependent AUC values compare the OS predictive ability **(E)** and RFS predictive ability **(J)** of gene signature and clinical predictors.

We next verified the prognostic performance of this signature in the TCGA validation cohort. The K–M curves estimated a remarkably shorter OS (**Figure 3A**) and RFS (**Figure 3F**) in patients with high-risk. Patients with a high-risk score had a significantly elevated mortality rate (**Figure 3B**) and recurrence rate (**Figure 3G**) compared with patients with a low-risk score. The calibration curves indicated that the predicted survival probability through this signature exhibited good consistency with the observed survival probability (**Figures 3C, H** **)**. The results of the univariate Cox regression analyses confirmed that the 8-gene signature and several clinical indicators were risk factors for OS (**Figure 3D**) and RFS (**Figure 3I**). Time-dependent AUC values further demonstrated that the 8-gene signature was not inferior to clinical parameters for OS prediction (**Figure 3E**) and RFS prediction (**Figure 3J**).

### Predictive Ability of the 8-Gene Signature and Previously Reported Signatures

**Figure 4:**
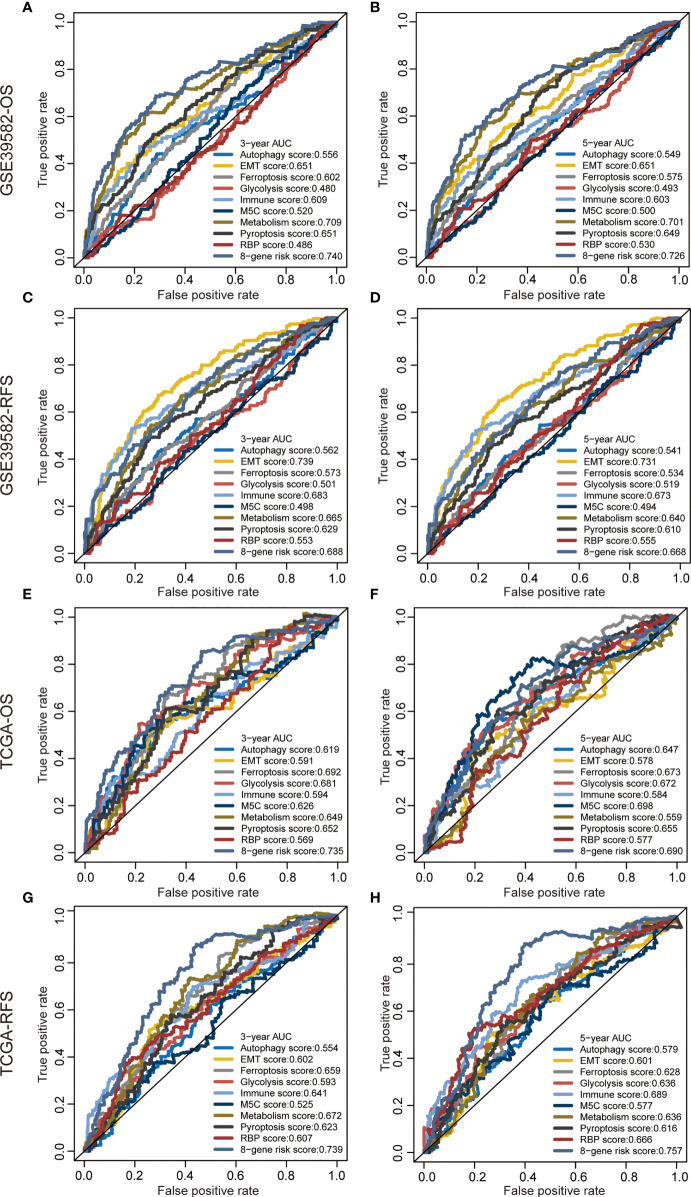
Predictive ability of the 8-gene signature compared with previous signatures. ROC curves of different prognostic signatures in predicting 3-year OS **(A)**, 5-year OS **(B)**, 3-year RFS, **(C)** and 5-year RFS **(D)** in the GSE39582 cohort, and 3-year OS **(E)**, 5-year OS **(F)**, 3-year RFS **(G),** and 5-year RFS **(H)** in the TCGA cohort.

We proved that the 8-gene signature outperformed clinical indicators regarding survival prediction. Through AUC value analysis, we subsequently compared the predictive ability of our signature with nine recently published signatures. The higher the AUC value is, the stronger the prediction ability is. Results showed that our gene signature had the highest predictive accuracy for 3-year OS prediction in the GSE39582 cohort (0.74, **Figure 4A**), 5-year OS prediction in the GSE39582 cohort (0.726, **Figure 4B**), 3-year OS prediction in the TCGA cohort (0.735, **Figure 4E**), 3-year RFS prediction in the TCGA cohort (0.739, **Figure 4G**), and 5-year RFS prediction in the TCGA cohort (0.757, **Figure 4H**). This gene signature had the second highest predictive accuracy for 3-year RFS prediction in the GSE39582 cohort (0.688, **Figure 4C**), 5-year RFS prediction in the GSE39582 cohort (0.668, **Figure 4D**), and 5-year OS prediction in the TCGA cohort (0.69, **Figure 4F**). These findings suggest that the 8-gene signature could provide an enhanced survival prediction for CRC patients.

### Nomogram Construction

**Figure 5:**
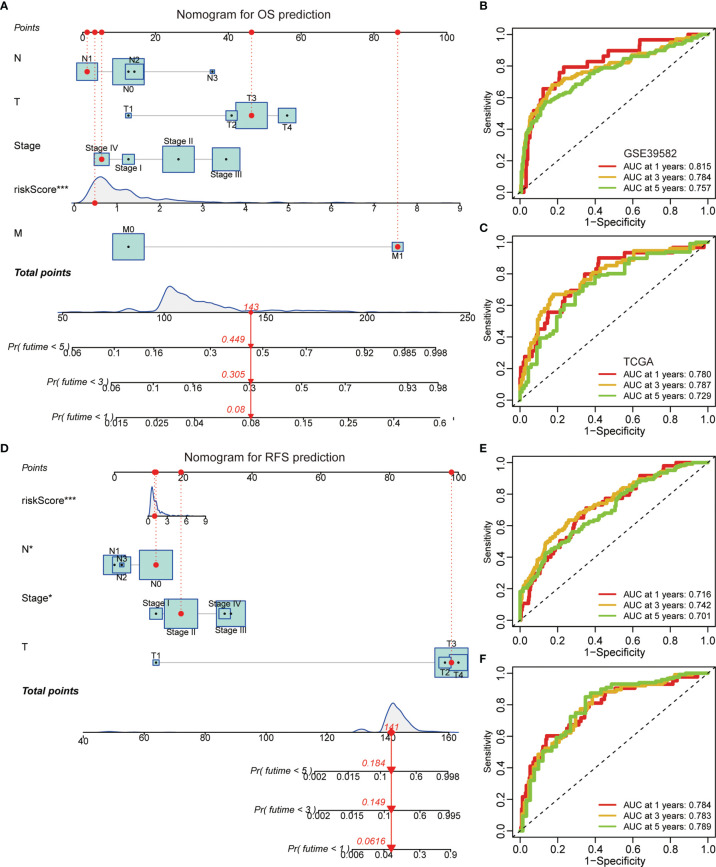
A nomogram based on the gene signature. **(A)** A nomogram integrating N stage, T stage, M stage, TNM stage, and risk score for OS prediction. **(B, C)** ROC curves of OS predictive nomogram in the GSE39582 **(B)** and TCGA **(C)** cohorts. **(D)** A nomogram integrating N stage, T stage, TNM stage, and risk score for RFS prediction. **(E, F)** ROC curves of RFS predictive nomogram in the GSE39582 **(E)** and TCGA **(F)** cohorts.

A graphic nomogram, namely, T stage, N stage, M stage, TNM stage, and risk score, was developed in the GSE39582 cohort to predict 1-, 3-, and 5-year OS (**Figure 5A**). ROC curves verified the high predictive accuracy (AUC value no less than 0.7) of this nomogram in the GSE39582 (**Figure 5B**) and TCGA (**Figure 5C**) cohorts. Similarly, a graphic nomogram integrating N stage, T stage, TNM stage, and risk score was constructed in the GSE39582 cohort to predict 1-, 3-, and 5-year RFS (**Figure 5D**). Following ROC curve analyses, further confirmation was obtained of the moderate accuracy of this nomogram for RFS prediction (**Figures 5E, F**).

### Biological Process and Pathway Enrichment Analyses of the 8-Gene Signature

**Figure 6:**
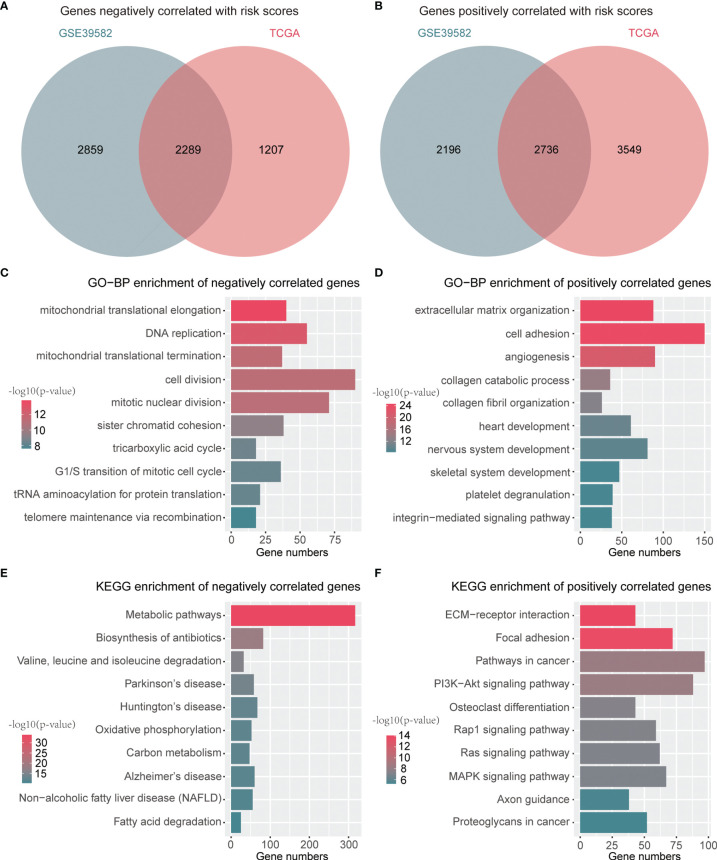
Functions and pathways related to the signature. **(A)** A Venn diagram for common genes with negative correlation to risk scores. **(B)** A Venn diagram for common genes with positive association with risk scores. **(C)** Top 10 GO-BP terms of genes negatively associated with risk scores. **(D)** Top 10 GO-BP terms of genes positively associated with risk scores. **(E)** Top 10 KEGG terms of genes negatively associated with risk scores. **(F)** Top 10 KEGG terms of genes positively associated with risk scores.

A total of 2,289 negatively correlated genes and 2,736 positively correlated genes with risk scores were identified through a Venn diagram (**Figures 6A, B**). These genes were then submitted for function annotation and pathway enrichment. For biological processes, negatively correlated genes were primarily involved in DNA replication, cell division, and the cell cycle (**Figure 6C**), whereas positively correlated genes were mainly associated with cell adhesion and angiogenesis (**Figure 6D**). For pathway enrichment, negatively correlated genes were primarily involved in metabolic pathways and oxidative phosphorylation (**Figure 6E**), while positively correlated genes were mainly related to PI3K–Akt signaling pathway, Rap1 signaling pathway, Ras signaling pathway, and MAPK signaling pathway (**Figure 6F**).

### CACNB1, HIST1H2BJ and INHBB Were Significantly Upregulated in CRC Tissues

**Figure 7:**
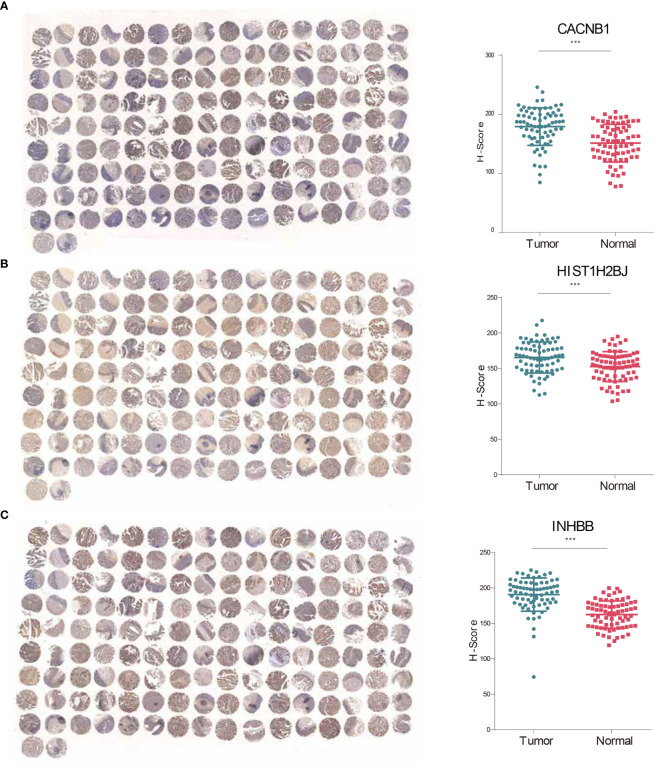
CACNB1, HIST1H2BJ, and INHBB were significantly upregulated in CRC tissues. **(A–C)** IHC staining and H-score of CACNB1 **(A)**, HIST1H2BJ **(B)**, and INHBB **(C)**, respectively. *p < 0.05; **p < 0.01; ***p < 0.001.

The expressive levels of three risky genes (Hazard ratio >1) in human CRC tissues and matched adjacent normal tissues were detected through IHC analyses. The results showed that CACNB1, HIST1H2BJ, and INHBB were significantly overexpressed in CRC tissues (**Figures 7A–C**). These findings suggest that CACNB1, HIST1H2BJ, and INHBB might play oncogenic roles in CRC development.

## Discussion

CRC is a lethal disease with high molecular heterogeneity that requires optimized treatment to prolong patient survival (32). Currently, the TNM staging system largely informs patient prognosis and treatment decisions. However, the suitability of this system for patients at the same stage is questionable because of intra-stage discrepancy caused by tumor heterogeneity (33). Therefore, acquiring effective prognostic biomarkers is critical to stratifying survival risk and tailor-specialized treatment. Thanks to significant advances in high-throughput sequencing and bioinformatics, prognostic gene signatures that translate tumor genetic and genomic features into a clinical application have emerged as a practical tool for survival prediction (34).

During the construction of the 8-gene risk signature, we initially identified and overlapped genes associated with both OS and RFS of CRC patients in two large cohorts. A total of 186 genes were screened, and the following LASSO regression analysis, together with multivariate Cox regression analysis, determined an optimal 8-gene signature. These eight genes had a minimal overlap with previous gene signatures. The K–M survival and calibration curves revealed that the signature could powerfully classify CRC patients with different outcomes. ROC analyses showed that the signature could provide better survival prediction than clinical predictors and previous models. A nomogram efficaciously predicts survival probabilities, strengthening the clinical applicability of the signature. Functional analyses suggest that the signature is positively associated with several oncogenic pathways, namely, cell division, cell adhesion, and DNA replication. In addition to the prognostic value, we observed that CACNB1, HIST1H2BJ, and INHBB were significantly upregulated in CRC tissues. These findings provide not only a supplement to the current TNM staging system for survival assessment but also multiple potential therapeutic targets and biomarkers for CRC.

Among the eight genes, four genes, namely, ATOH1, CACNB1, EPHB2, and IHNBB, are reported to be involved in CRC tumorigenesis. ATOH1 is frequently downregulated and plays a tumor suppressive role in CRC (35). It serves as a novel factor downstream of the Wnt pathway that is capable of suppressing anchorage-independent growth of colon cancer cell lines (36). As EPHB2 is also a tumor suppressor gene for CRC, it is downregulated in CRC tissues, and low levels of EPHB2 expression are associated with a shorter mean duration of survival (37, 38). *In vitro* biological studies demonstrated that overexpression of EPHB2 inhibited CRC cell proliferation and migration (39). INHBB is a novel prognostic biomarker and its overexpression in CRC tissues indicates a poor prognosis (40). Additionally, the overexpression of INHBB is significantly positively correlated with the depth of invasion, distant metastasis, and CRC stage (41). Similar to INHBB, elevated CACNB1 expression in CRC is associated with poor patient survival (42). The other four genes also have some tumor-specific functions, but their biological roles in CRC remain largely unknown.

Considerable progress in bioinformatics and high-throughput sequencing enables the novel development of prognostic models in human cancers (43). In CRC, many powerful gene signatures have been established to predict OS or RFS, while risk models for both OS and RFS prediction are rare (44). This study is the first to establish OS and RFS prediction models for CRC patients *via* credible prognostic genes. Stratifying CRC patients according to the predicted survival probability and recurrence risk may facilitate individual therapy and surveillance imaging. Validation in the two largest CRC cohorts, including American and European populations, reinforces the reliability of this signature. We hope that this model can be transformed into a PCR-based rapid detection kit. It may offer potential value for saving public health resources and for exempting patients from the heavy financial burden and unnecessary cytotoxicity of overtreatment.

However, there are still many limitations to this study. First, this signature is based on retrospective data, and needs to be verified in more prospective cohorts. Second, the cohorts enrolled in this study are relatively small, so this signature needs further validation in more large-sized cohorts in the future. Third, tumor infiltrative immune cells and immune-related genes have been proved to play critical roles in the development and progression of tumors (45), but there are only minor intersected differences in immune cell infiltration between low-risk and high-risk groups in both training and validation cohorts (data not shown). Furthermore, we have only preliminarily experimentally verified the abnormal expression of CACNB1, HIST1H2BJ, and INHBB without exploring their biological functions. Therefore further *in vivo* and *in vitro* experiments are needed to illuminate their potential functions in CRC progression.

In conclusion, we proposed a novel gene signature for both OS and RFS prediction and confirmed the efficient predictive ability of this signature. The risk signature is beneficial for increasing treatment precision and maximizing survival benefit and quality of life. After all, this signature was based on retrospective cohorts and needed to be further validated in more prospective cohorts.

## Data Availability Statement

The original contributions presented in the study are included in the article/**Supplementary Material** Further inquiries can be directed to the corresponding authors.

## Ethics Statement

Written informed consent was obtained from all patients. This study was conducted and approved in accordance with the Declaration of Helsinki, and the Ethics Committee of Shanghai Changhai Hospital approved the study.

## Author Contributions

WZ designed the study and drafted the manuscript. LZ, YY, and GY prepared the tables and figures and drafted the manuscript. RW, KZ, SJ, XZ, SJ, and HG contributed to the clinical sample collection. ZL and LH contributed to the editing and review. All authors listed have made a substantial, direct, and intellectual contribution to the work and approved it for publication.

## Funding

This work was supported by the National Natural Science Foundation of China (82072750), the Natural Science Fund of Shanghai (20ZR1457200), and the Shanghai Sailing Program (21YF1459300).

## Conflict of Interest

The authors declare that the research was conducted in the absence of any commercial or financial relationships that could be construed as a potential conflict of interest.

## Publisher’s Note

All claims expressed in this article are solely those of the authors and do not necessarily represent those of their affiliated organizations, or those of the publisher, the editors and the reviewers. Any product that may be evaluated in this article, or claim that may be made by its manufacturer, is not guaranteed or endorsed by the publisher.

## Abbreviations

AIC, Akaike Information Criterion; AUC, area under the curve; CRC, colorectal cancer; GEO, Gene Expression Omnibus; GO-BP, gene ontology-biological process; IHC, immunohistochemical; KEGG, Kyoto Encyclopedia of Genes and Genomes; K–M, Kaplan–Meier; LASSO, least absolute shrinkage and selection operator; OS, overall survival; ROC, receiver operating characteristic; RFS, recurrence-free survival; TCGA, The Cancer Genome Atlas; TNM, tumor, node, metastasis.
